# How to check eye alignment and movement

**Published:** 2019-12-17

**Authors:** Eugene Helveston, Anand Moodley

**Affiliations:** 1 Global director: ORBIS Cyber-Sight, ORBIS International, New York, USA.; 2 Head of Clinical Department: Neurology, Universitas Academic Hospital, University of the Free State, Bloemfontein, South Africa.

**If a child's eyes are not lined up correctly, then the vision in the deviated eye may be reduced permanently. Early detection and referral is essential.**

In order to achieve normal binocular vision, the eyes must see well, be aligned (i.e., look in the same direction), and be focused on the same object. To maintain alignment, the eyes must also move in a coordinated manner.

Misalignment of the eyes is called **strabismus** (or **squint**). Misalignment means that the eyes are not lined up to look at the same thing. In strabismus or misalignment, one eye is fixed on what the person intends to look at (the fixing eye) and the other eye is looking at something else (the deviated eye). In young children the brain tends to suppress the image in the deviated eye, while in adults a new squint (misalignment) can cause double vision. If a child has strabismus from a young age and is not treated, the vision in the deviated eye can become permanently reduced; this is called **amblyopia** or ‘lazy eye’.

It is therefore very important to detect strabismus as early as possible and to refer the patient to an ophthalmologist or other relevant eye care professional.

## Step 1. Check ocular alignment using a torch

**Figure 1:**
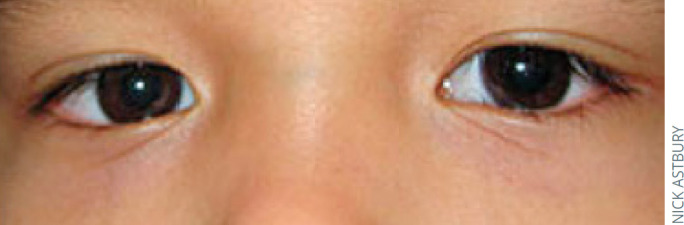
Straight eyes. The light reflex is seen in the centre of the pupils in both eyes. Although this boy's eyes tend to look crossed (because he is looking slightly to the left and the bridge of his nose is broad), the light reflexes in the centre of his pupils confirm that his eyes are straight and aligned.

Check the alignment of the eyes. This is performed by comparing the light reflex from the cornea of both eyes. Hold a torch 1 metre in front of the eyes and look for the light reflex on the cornea (Hirschberg test). In the primary gaze (looking straight ahead at the torch light), the light reflexes should be in a symmetrical position on each cornea (Figure 1).

**Figure 2:**
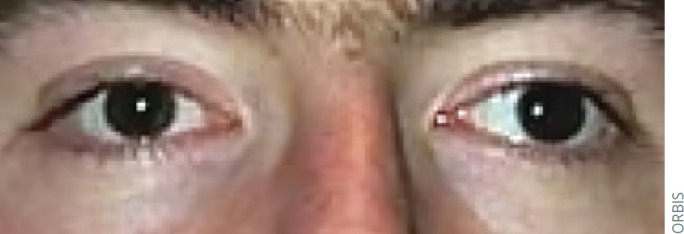
Exotropia in the left eye. The light reflex is central in the right eye and over the iris in the left eye.

**Figure 3:**
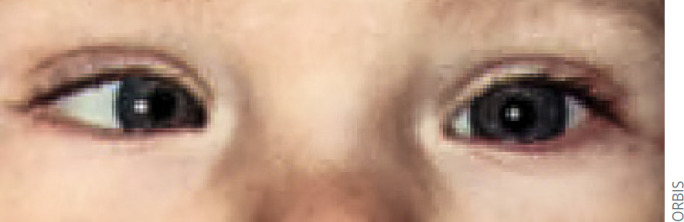
Esotropia in the right eye. The light reflex is central in the left eye and over the iris in the right eye.

If one eye is turning **out**, this is called exotropia (Figure 2), whereas if the eye is turning **in** it is called esotropia (Figure 3).

## Step 2. Check for abnormal head posture

Look at the patient and see if they hold their head in an abnormal position. In some instances, the person with a strabismus assumes an abnormal position of the head to try to keep the eyes aligned. For example, the child or adult will turn their head or raise or lower the chin to help the eyes to become aligned with what they are looking at.

## Step 3. Do the cover test

If you find that an eye is misaligned, use the cover test to confirm this. For example, say that you have observed the right eye turning in when the patient looks straight ahead (as in Figure 3). If you then cover the left eye (the normal eye), you should see the right eye (the deviated eye) turn towards the front. This confirms that the right eye was not aligned with the left eye when both eyes were open.

## Step 4. Check ocular movements and double vision

**Figure 4:**
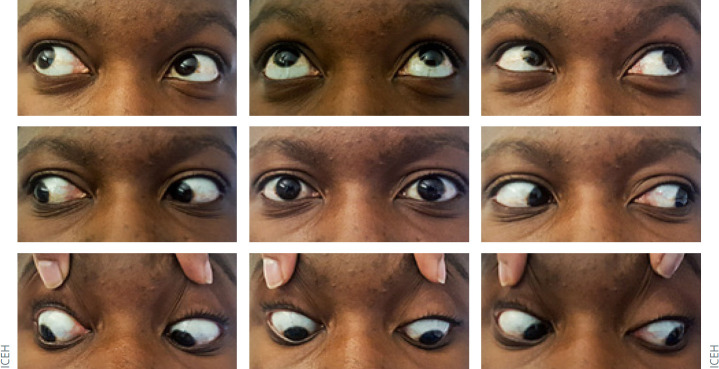
The nine positions of gaze in someone with healthy eyes

When checking a person for strabismus, it is necessary to confirm that the eyes can move freely in all directions. There are nine possible positions of gaze, as shown in Figure 4. Check eye movements by holding the patient's head still and asking him or her to follow your finger or a light as you move it from looking in front to each of the nine positions in turn. Note any limitation of movement of one or both eyes.

If a patient complains of double vision (diplopia) then while checking ocular movements, ask the patient if they see one or two torch images in each position of gaze. The gaze of maximal double vision can help identify which muscle and nerve is not working.
